# Dasatinib-Associated Recurrent Symptomatic Bilateral Pleural Effusions in an Elderly Patient With End-Stage Renal Disease on Maintenance Hemodialysis

**DOI:** 10.7759/cureus.99571

**Published:** 2025-12-18

**Authors:** Macaulay A Onuigbo, Margaret Butler, Jessica Biediger

**Affiliations:** 1 Medicine, The Robert Larner, M.D. College of Medicine, University of Vermont, Burlington, USA

**Keywords:** bilateral pleural effusions, chronic myeloid leukemia (cml), dasatinib, intradialytic parenteral nutrition, maintenance hemodialysis

## Abstract

Dasatinib, a second-generation tyrosine kinase inhibitor, is effective in treating chronic myeloid leukemia but can occasionally cause pleural effusions, even after long-term therapy. We present the case of an 87-year-old male with end-stage renal disease (ESRD) on thrice-weekly maintenance hemodialysis who, over eight months in 2025, required repeated bilateral therapeutic thoracenteses for recurrent symptomatic pleural effusions. These effusions developed more than seven years after initiating dasatinib, raising the question of whether they were secondary to dasatinib, ESRD-associated hypervolemia, or newly diagnosed pulmonary hypertension.

## Introduction

Dasatinib is a second-generation tyrosine kinase inhibitor (TKI) used in the treatment of chronic myeloid leukemia (CML) [1,2]. In Phase III clinical trials, pleural effusions were reported as an adverse reaction in up to 6% of patients and, in some cases, occurred after many years of therapy [3]. These effusions sometimes necessitated drug discontinuation [1-3]. Early recognition of symptoms is essential for proper management, and prompt diagnosis and intervention can minimize morbidity while preserving the long-term clinical benefits of dasatinib [1-3]. Older age and higher drug levels have been associated with more severe pleural effusions [4].

We present the case of an 87-year-old male with end-stage renal disease (ESRD) on maintenance hemodialysis since 2015 who, by mid-2025, had required repeated bilateral therapeutic thoracenteses over the preceding eight months for recurrent symptomatic bilateral pleural effusions, despite regular thrice-weekly hemodialysis and without excessive interdialytic weight gain. The question arose whether these effusions were secondary to ESRD-associated hypervolemia, newly evident pulmonary hypertension on echocardiography, or dasatinib-associated adverse reactions, even though the patient had been on the same dose of the TKI since 2017.

## Case presentation

An 87-year-old male with oligoanuric ESRD on thrice-weekly maintenance hemodialysis since 2015 and a medical history of atrial fibrillation with heart failure and preserved ejection fraction was diagnosed in November 2007 with CML, with an initial white blood cell count of 373.6 K/µL (reference range: 4.0-10.4 K/µL). He initially responded to imatinib, a first-generation TKI, and remained in remission through 2016-2017. He experienced a recurrence in early 2017 and was subsequently started on dasatinib, 20 mg daily, a second-generation TKI.

At diagnosis in November 2007, CML was characterized by 1% blasts in the bone marrow, a white blood cell count as high as 373.6 K/µL, and leukemic retinopathy. PCR for BCR/ABL was 88.6%, and cytogenetics showed a single (9;22) translocation in 20/20 cells, with 93.5% positivity in peripheral blood by fluorescence in situ hybridization (FISH). Imatinib 400 mg daily was initiated on November 8, 2007. Hematologic remission was achieved in January 2008 following a cytopenic interval in late December 2007. FISH became negative for BCR/ABL, and PCR was 0.069% in May 2008, consistent with a major molecular response. Imatinib was maintained at 200 mg daily and increased to 300 mg daily in December 2008 due to rising BCR/ABL PCR levels. He remained in remission with close hematology follow-up until March 2016, when intractable nausea and vomiting necessitated discontinuation of imatinib.

In early 2017, while in Florida, BCR/ABL transcript levels increased to 0.79%, and dasatinib 20 mg daily was started in June 2017. The dose was reduced to 20 mg three times weekly in July 2017 due to gastrointestinal side effects and was taken on Tuesdays and Fridays. Starting in October 2022, the dose schedule was adjusted to 20 mg on Mondays, Wednesdays, and Fridays after dialysis.

**Figure 1:**
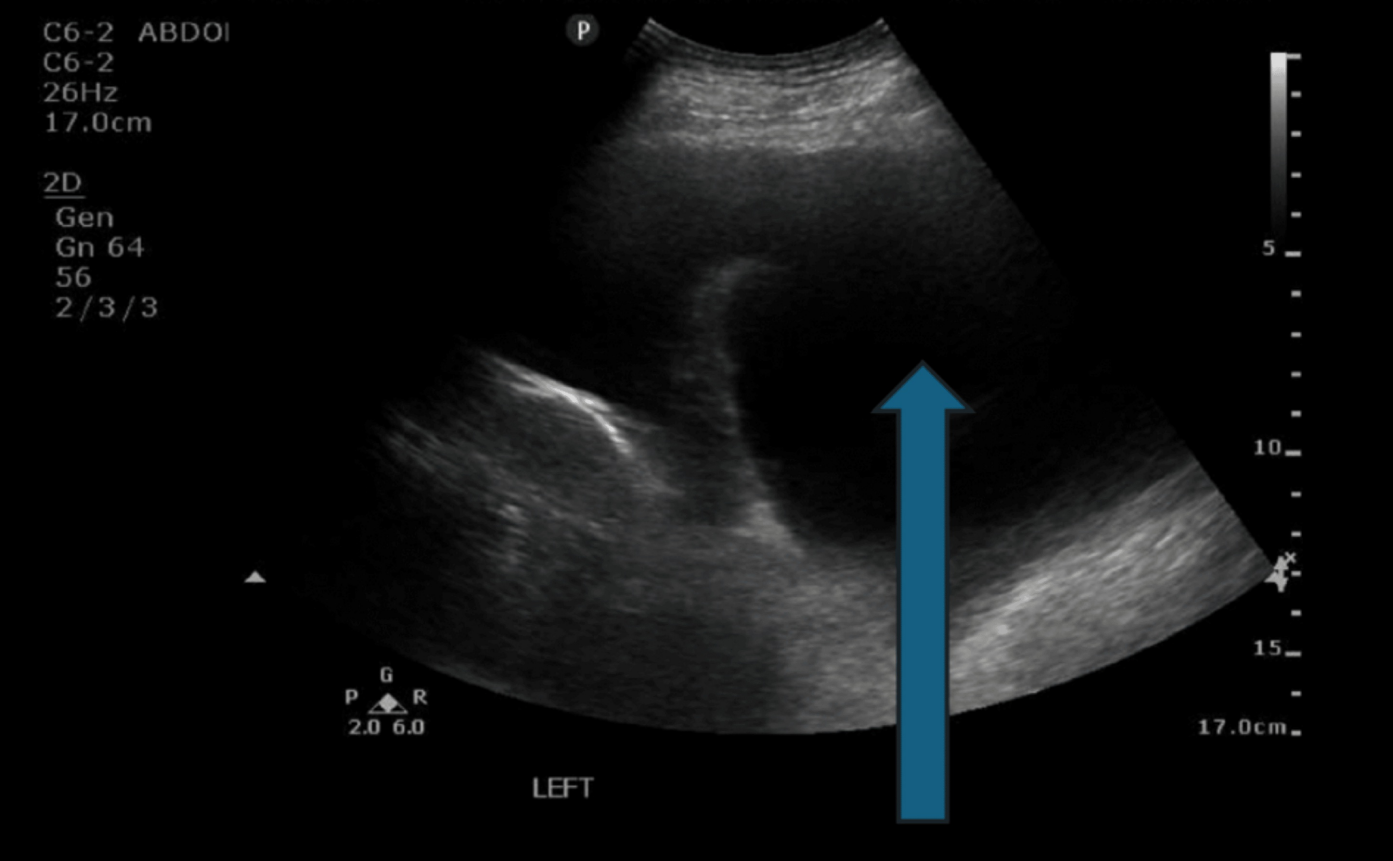
Left pleural effusion evident on ultrasound-guided thoracentesis in November 2024

The patient generally tolerated hemodialysis well and, in recent years, dialyzed via a left thigh arteriovenous graft. In late 2021, intradialytic parenteral nutrition (IDPN) was initiated for malnutrition with hypoalbuminemia. In March 2022, he developed right-sided community-acquired pneumonia complicated by a right pleural effusion, which required therapeutic thoracentesis of 1.5 liters of serosanguinous fluid with a total protein of 3.4 g/dL. This was considered a parapneumonic effusion and did not recur after treatment. Over the subsequent 32 months, he continued on maintenance thrice-weekly hemodialysis with thrice-weekly IDPN supplementation. Symptomatic bilateral pleural effusions developed for the first time in late November 2024 (Figure 1).

He required ultrasound-guided bilateral therapeutic thoracentesis on multiple occasions between November 2024 and July 2025, with varying volumes of pleural fluid removed from both lungs, resulting in symptomatic relief (Table 1).

**Table 1:** Volumes and descriptions of pleural fluid removed in four procedures during 2024-2025

Date of bilateral therapeutic thoracentesis	November 26, 2024	May 20, 2025	July 7, 2025	July 28, 2025
Fluid removed from the right pleural space	500 mL yellow fluid	1,400 mL clear yellow fluid	1,300 mL clear yellow fluid	1,200 mL clear yellow fluid
Fluid removed from the left pleural space	1,500 mL yellow fluid	600 mL clear yellow fluid	1,050 mL tea-colored fluid	1,000 mL amber fluid

A subsequent echocardiogram in March 2025 revealed a left ventricular ejection fraction (LVEF) of 55-60%, a normal-sized right atrium, and a normal-sized inferior vena cava with a normal right atrial pressure of 0-5 mm Hg. Pulmonary hypertension was noted, with a pulmonary artery pressure ≥50 mm Hg, and no pericardial effusion was observed.

**Figure 2:**
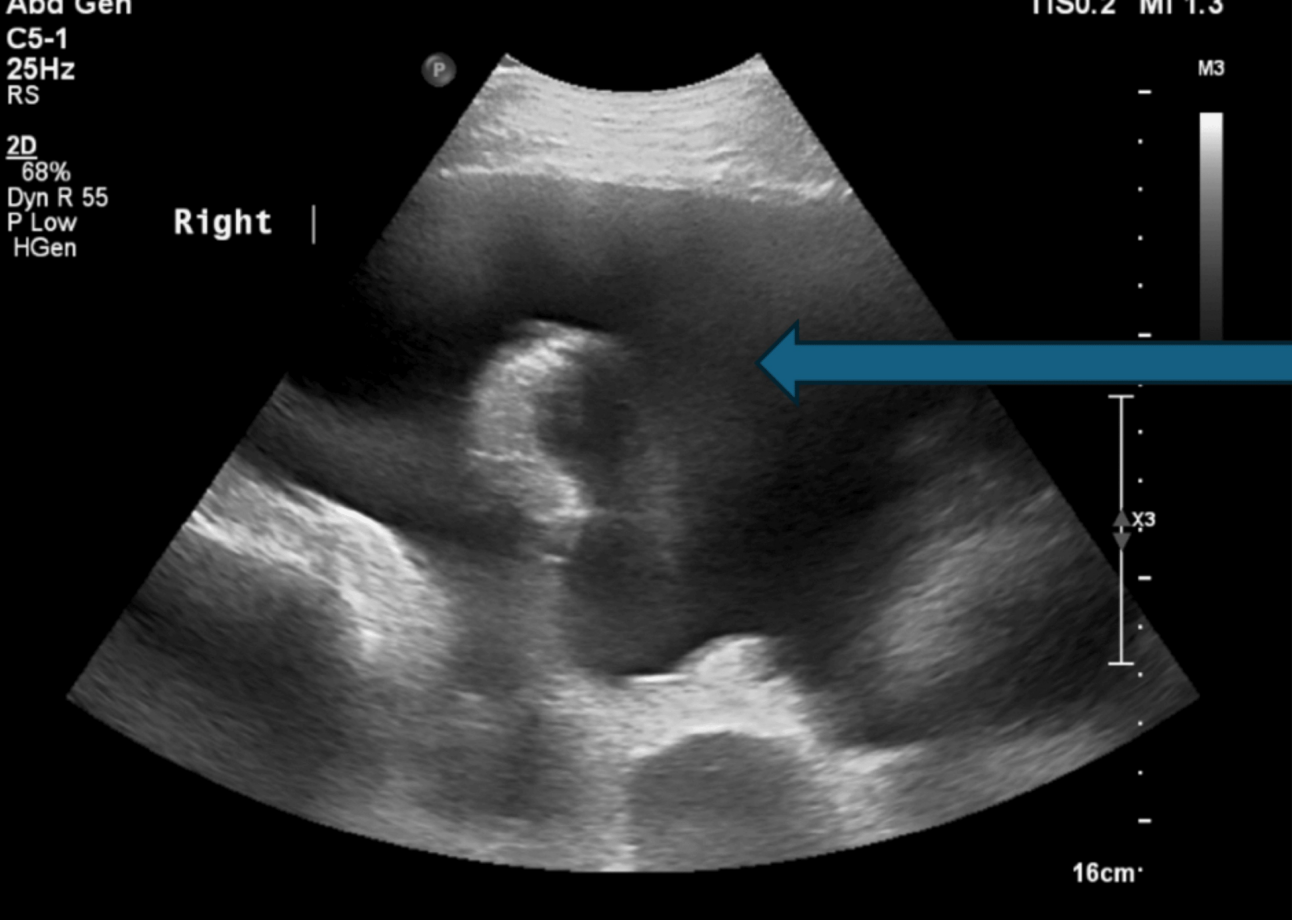
Right pleural effusion evident on ultrasound-guided thoracentesis in May 2025

**Figure 3:**
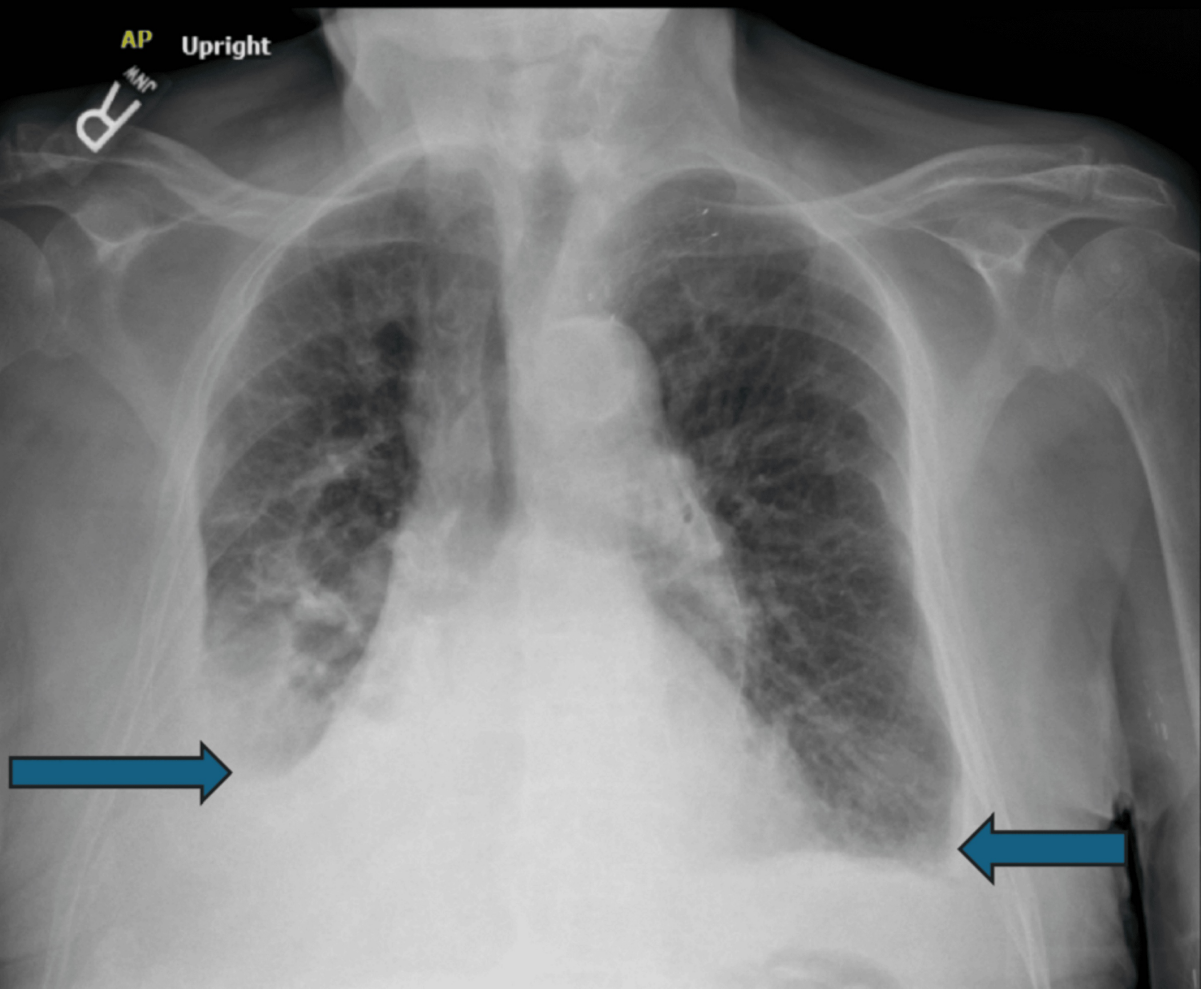
Chest radiograph in May 2025, one day before the ultrasound-guided bilateral thoracentesis

**Figure 4:**
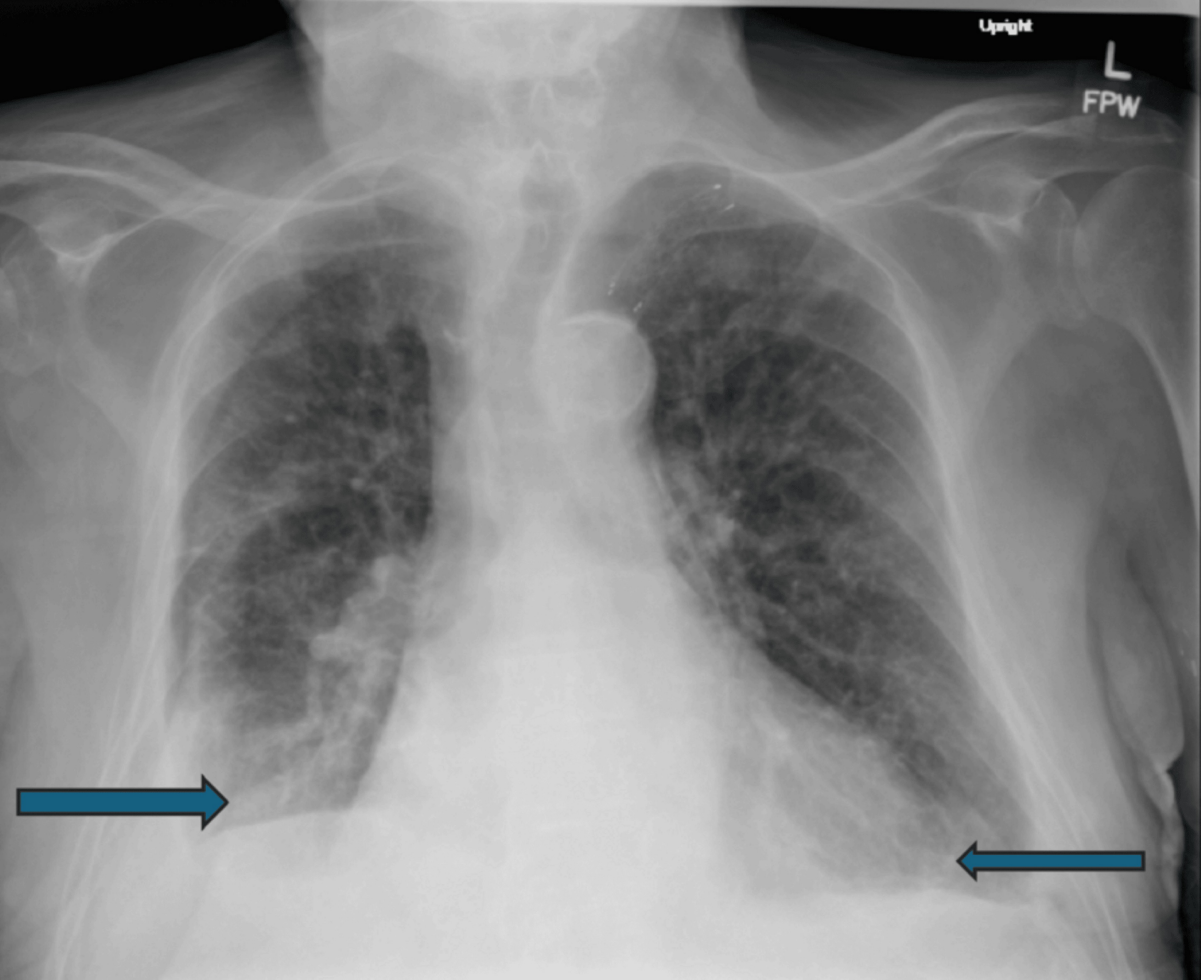
Chest radiograph in early July 2025, two days after bilateral therapeutic thoracentesis

Since then, he required repeated ultrasound-guided bilateral therapeutic thoracentesis for recurrent symptomatic bilateral pleural effusions in May 2025 (Figure 2, Figure 3), again in early July 2025 (Figure 4), and once more in late July 2025. Notably, NT-proBNP levels increased to 47,500 pg/mL in May 2025 and further to 91,300 pg/mL in July 2025. Efforts to achieve higher net ultrafiltration during hemodialysis to prevent recurrence were unsuccessful. At each episode, chest radiographs confirmed the recurrent bilateral effusions (Figure 3, Figure 4). The volumes of pleural fluid removed during all four thoracentesis procedures are summarized in Table 1.

In late July 2025, he was admitted to the hospital with persistent chills, malaise, and altered mental status, one day after the fourth therapeutic bilateral thoracentesis. At that time, dasatinib was strongly suspected to be the primary cause of the recurrent bilateral pleural effusions and was therefore suspended. Simultaneously, oxybutynin, which had been started approximately six weeks earlier for painful dysuria, was discontinued as it was suspected of contributing to the altered mental status. His mental status promptly returned to baseline thereafter.

Since the discontinuation of dasatinib, he has done very well, with normalized breathing and no new shortness-of-breath symptoms for four months through November 2025. He has continued thrice-weekly in-center maintenance hemodialysis with a stable prescribed target weight. Four months after dasatinib was discontinued, he remains in good spirits, is very active, and has had no recurrence of bilateral pleural effusions.

Dasatinib was formally discontinued in early August 2025 due to recurrent moderate-to-severe symptomatic bilateral pleural effusions. Hematology has continued to follow him closely. At his last hematology visit in early December 2025, BCR-ABL for the P210 transcript was detected at 0.3%, with a repeat test pending from early December 2025. Hematology plans to continue observation if the BCR-ABL level remains below 1%. If it exceeds 1.0%, a change to another agent, such as bosutinib or asciminib, is planned. Monthly BCR-ABL testing for the P210 transcript is scheduled.

## Discussion

With the advent of BCR-ABL1 TKIs, CML has become a manageable disease, with the possibility of near-normal life expectancy [1,2]. Dasatinib, a second-generation TKI, is effective in treating CML [1,2]. Pleural effusions have been reported in up to 6% of patients in Phase III clinical trials and can occur after many years of therapy [3]. In these trials, pleural effusions sometimes necessitated drug discontinuation [1-3]. Early identification of symptoms is essential for proper management. Prompt diagnosis and treatment can minimize morbidity and help preserve the long-term clinical benefits of dasatinib [1-3]. Older age and higher drug levels have been associated with more severe pleural effusions [4].

Discontinuation of dasatinib due to pleural effusions was necessary in a minority of patients with chronic-phase CML (CML-CP) in both the DASatinib versus Imatinib Study in Treatment-Naïve CML Patients (DASISION) trial [1,5] and the CA180-034 study, a Phase III dose-optimization trial evaluating dasatinib dosing regimens in imatinib-resistant or -intolerant CML-CP patients [6].

Medium to large pleural effusions typically require therapeutic thoracentesis, and other etiologies must be ruled out [1,5,6]. Importantly, pleural effusions can occur at any time during dasatinib therapy. For example, in DASISION, the median time to first grade 1/2 pleural effusion was 114 weeks (range: 4-299 weeks) [1,5]. Therefore, all patients receiving dasatinib require careful monitoring, and any effusions that occur must be promptly managed [1,5].

Given the rapid recurrence of symptomatic bilateral effusions in our patient in 2025, with no evidence of significant volume overload (stable weight and physical exam), and although pulmonary hypertension may have contributed, as indicated by echocardiographic data, we believe the recurrent bilateral pleural effusions were more likely secondary to dasatinib, even after several years of therapy. This conclusion is strongly supported by the absence of any recurrence through the end of October 2025, three months after dasatinib was discontinued.

There was no evidence of severe decompensated right ventricular failure, such as worsening symptomatic right heart failure from pulmonary hypertension. The patient did not exhibit hepatomegaly, a pulsatile or tender liver, new peripheral edema, or ascites. Interdialytic weight gains remained stable, and he continued to tolerate thrice-weekly IDPN infusions for nutritional support.

Finally, dasatinib is known to cause pulmonary hypertension. Echocardiograms performed in March and July 2025, during the period of recurrent symptomatic bilateral pleural effusions, showed stable LVEF (50-55%) and normal right ventricular function, with mild-to-moderately elevated pulmonary artery systolic pressure (45 to ≥50 mm Hg). A much earlier echocardiogram from October 2021 reported LVEF of 50-55% with normal right ventricular function, though pulmonary artery systolic pressure was not reported.

Given that the patient’s dyspnea promptly resolved following bilateral therapeutic thoracentesis, pleural effusions have not recurred for over four months, and pulmonary artery systolic pressure remained stable between March and July 2025, the current management approach is conservative monitoring of pulmonary hypertension. It remains unclear whether the pulmonary hypertension was secondary to long-term dasatinib therapy.

## Conclusions

Under continued clinical surveillance since the discontinuation of dasatinib in late July 2025, we conclude that our patient’s recurrent symptomatic bilateral pleural effusions, despite repeated, nearly monthly bilateral therapeutic thoracenteses, with 0.5-1.5 liters of pleural fluid removed from each lung, were secondary to dasatinib adverse reactions. As reported in previous Phase III trials, such pleural effusions can occur after several years of therapy. Our patient started dasatinib for CML in early 2017 and only began exhibiting recurrent pleural effusions in November 2024, over seven years later. Four months after dasatinib was discontinued, he has remained in good spirits, is very active, experiences no shortness of breath, and has had no recurrence of bilateral pleural effusions.

Coincidentally, the patient’s altered mental status developed about six weeks after starting oxybutynin and resolved promptly after discontinuation during the late July 2025 admission. This case underscores the importance for clinicians of considering both the timing and potential causality of adverse drug reactions. In this patient, altered mental status developed early, several weeks after initiation of oxybutynin, whereas recurrent bilateral pleural effusions secondary to dasatinib only emerged after nearly seven years of therapy.
